# Accessory Articulation of Elongated Anterior Tubercles of the Transverse Processes at C5-C6 in a Young Adult Female Patient: A Case Report

**DOI:** 10.7759/cureus.108902

**Published:** 2026-05-15

**Authors:** Britta E Verhey, Joshua R Sokol

**Affiliations:** 1 Anatomy, University of Nevada Reno School of Medicine, Reno, USA; 2 Radiology, Virtual Radiologic, Eden Prarie, USA

**Keywords:** accessory articulation, anatomical variant, cervical spine, computed tomography, congenital variant, teleradiology, transverse process, tubercle

## Abstract

Elongation of the anterior tubercle of the cervical transverse process with formation of an accessory articulation between adjacent vertebrae is a rare anatomical variant. Recognition of this variant is important to avoid misidentification as an acute fracture fragment in the trauma setting. In this report, a 22-year-old female patient presented to the emergency department following a motor vehicle collision (MVC). Per patient self-report, there was no known prior cervical spine pathology or previously identified congenital anomalies. Non-contrast computed tomography (CT) of the cervical spine with multiplanar reformatted images in the axial, coronal, and sagittal planes and three-dimensional surface-shaded display (SSD) reconstructions revealed no acute fracture, subluxation, or listhesis. An incidental finding demonstrated unilateral elongated left C5-C6 anterior tubercles of the transverse processes with a well-corticated accessory articulation between them. The articulation exhibited smooth, sclerotic articular surfaces morphologically consistent with a synovial-type articulation, although histological confirmation was not available. Transverse foramina were patent bilaterally without osseous narrowing. Vertebral artery patency was not directly assessable on this non-contrast examination. No cervical rib was identified at C7 on either side. This case was interpreted via teleradiology with limited access to clinical history, prior imaging, and follow-up. Recognition of this rare anatomical variant is important for radiologists and emergency physicians to avoid misidentification as an acute fracture fragment in the trauma setting. Well-corticated margins and sclerotic articular surfaces distinguish this congenital variant from fracture fragments. CT with multiplanar reformations provides detailed characterization of this variant.

## Introduction

The anterior tubercle of the cervical transverse process represents the vestigial costal elements at cervical levels and is a well-recognized anatomical landmark for surgical approaches and radiological interpretation [1]. Each cervical transverse process (C3-C6) consists of an anterior and posterior tubercle connected by a costotransverse bar, forming the boundaries of the transverse foramen through which the vertebral artery ascends. This finding is classified as a congenital anatomical variant, placing it within the spectrum of normal human anatomical diversity, rather than a congenital anomaly, which implies deviation from normal with potential pathological significance. First described in 1983 by Applbaum et al., elongation of the anterior tubercles of the cervical transverse process with accessory articulation formation is a rare anatomical variant [2]. This rare variant may be misidentified as an acute fracture fragment during trauma evaluation [3]. Recognition of these variants is fundamental to accurate radiological interpretation, particularly in the trauma setting where misidentification as a fracture can lead to unnecessary immobilization, additional imaging, or surgical consultation [3].

A systematic search of PubMed and Google Scholar was performed through May 2026 using the following search terms ("anterior tubercle" OR "anterior transverse process" OR "costal element" OR "cervical transverse process elongation" OR "hypertrophic anterior tubercle") AND ("accessory articulation" OR "accessory joint" OR "pseudoarticulation" OR "anatomical variant" OR "elongation" OR "incomplete segmentation"). Reference lists of all identified articles were manually reviewed. Cases were included if they described elongation of the anterior tubercle of the cervical transverse process with the formation of a discrete accessory articulation between adjacent C5 and C6 vertebrae. Using these criteria, five prior cases with confirmed or probable articulation formation were identified [2,4-7]. Additional reports of elongated anterior tubercles at other cervical levels or without confirmed articulation represent a related spectrum of costal element variants [8-10]. Cervical ribs arising from C7 are a far more common costal element variant, with a reported prevalence of 0.58%-6.2% depending on the population [11,12], and a pooled prevalence of approximately 1.1% in a meta-analysis [13].

This study was exempt from institutional review board (IRB) review, as it involves a single case report with no experimental intervention. No informed consent was required, as all pertinent history and images were anonymized and de-identified prior to publication. This case report was prepared in accordance with the CAse REport (CARE) guidelines.

We report the case of a 22-year-old female presenting following a motor vehicle collision (MVC) in whom non-contrast computed tomography (CT) of the cervical spine, interpreted by teleradiology, incidentally revealed this rare anatomical variant. This case expands the limited demographic spectrum of reported cases and reinforces the importance of distinguishing well-corticated accessory articulations from acute fracture fragments during trauma evaluation.

## Case presentation

A 22-year-old female patient presented to the emergency department following an MVC (no further details regarding the specific mechanism were available). Per patient self-report, there was no known prior cervical spine pathology or previously identified congenital anomalies. No prior cervical spine imaging was available for comparison. The patient reported generalized pain without focal cervical spine tenderness, neurological symptoms, or extremity complaints. This case was interpreted via teleradiology with limited access to detailed clinical history, physical examination findings, prior imaging, and patient follow-up. Clinical information was restricted to that provided in the imaging requisition.

**Figure 1:**
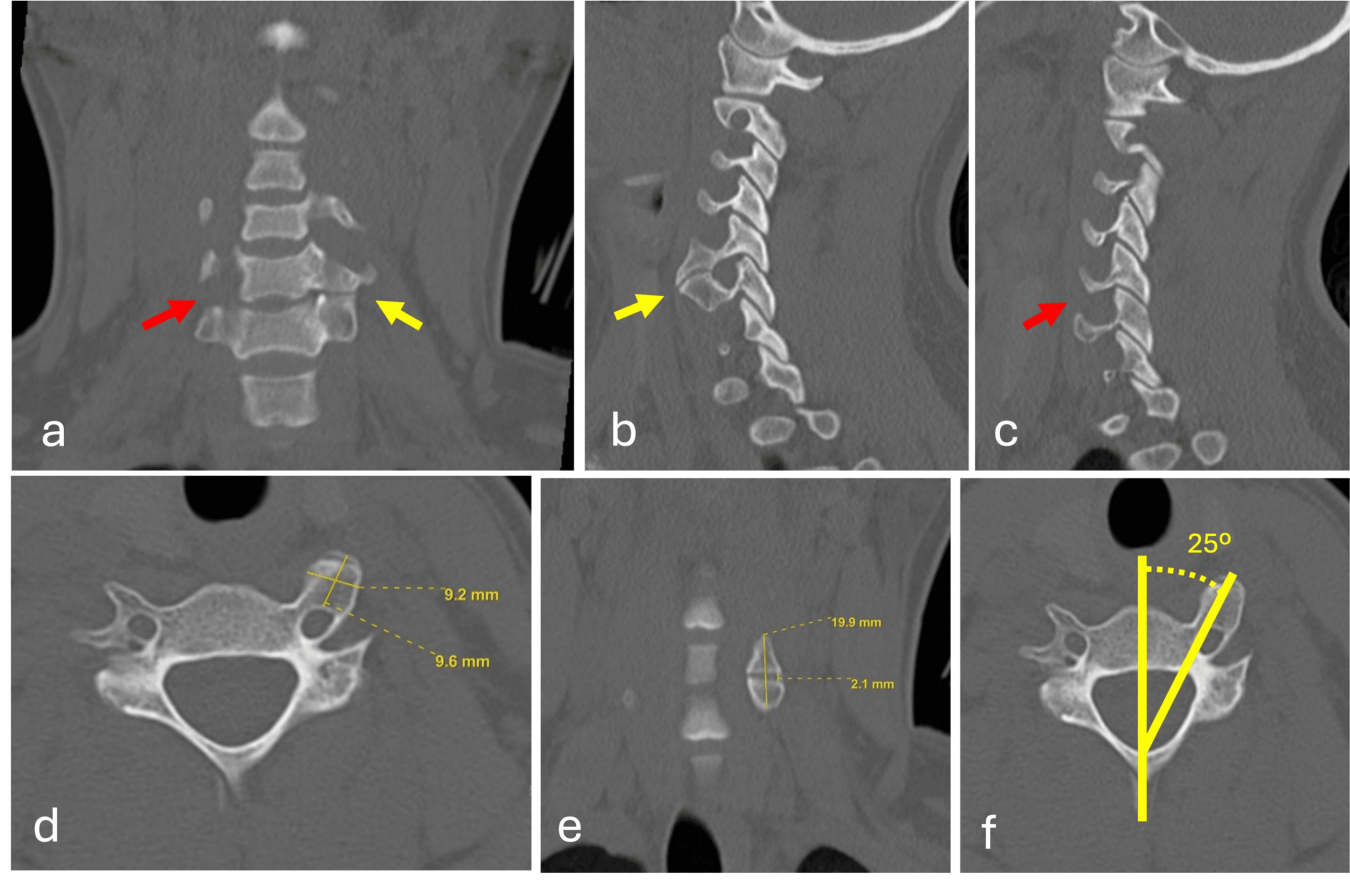
Multiplanar CT images demonstrating accessory articulation of elongated anterior tubercles of the left transverse processes at C5-C6. (a) Coronal reformatted CT image demonstrates asymmetric elongation of the anterior tubercles of the left C5 and C6 transverse processes forming a well-corticated accessory articulation (yellow arrow). The normal right-sided anterior tubercles of the C5 and C6 transverse processes are indicated for comparison (red arrow). No acute fracture or subluxation is identified. (b) Left parasagittal reformatted CT image shows the elongated anterior tubercles of the C5 and C6 transverse processes with a smooth, well-corticated accessory articulation between them (yellow arrow). (c) The right parasagittal reformatted CT image demonstrates normal morphology of the right C5 and C6 anterior tubercles of the transverse processes (red arrow), providing contralateral comparison to the left-sided variant. (d) Axial CT image at the C5–C6 level demonstrates the elongated left anterior tubercle of the transverse process projecting anterolaterally. Annotated measurements show the elongated anterior tubercle length (9.2 mm) and the total anteroposterior transverse process dimension (9.6 mm). The left transverse foramen is patent. Note the normal morphology of the right transverse process for comparison. (e) Coronal reformatted CT image with annotated measurements demonstrates the craniocaudal length of the combined elongated anterior tubercles and accessory articulation (19.9 mm) and the joint space width (2.1 mm) at the articulation. (f) Axial CT image with annotated measurement demonstrates the angle of projection of the elongated left anterior tubercle relative to the sagittal vertebral body axis (25°). The anterolateral projection is directed away from the expected course of the cervical nerve roots and vertebral artery within the transverse foramen.

**Figure 2:**
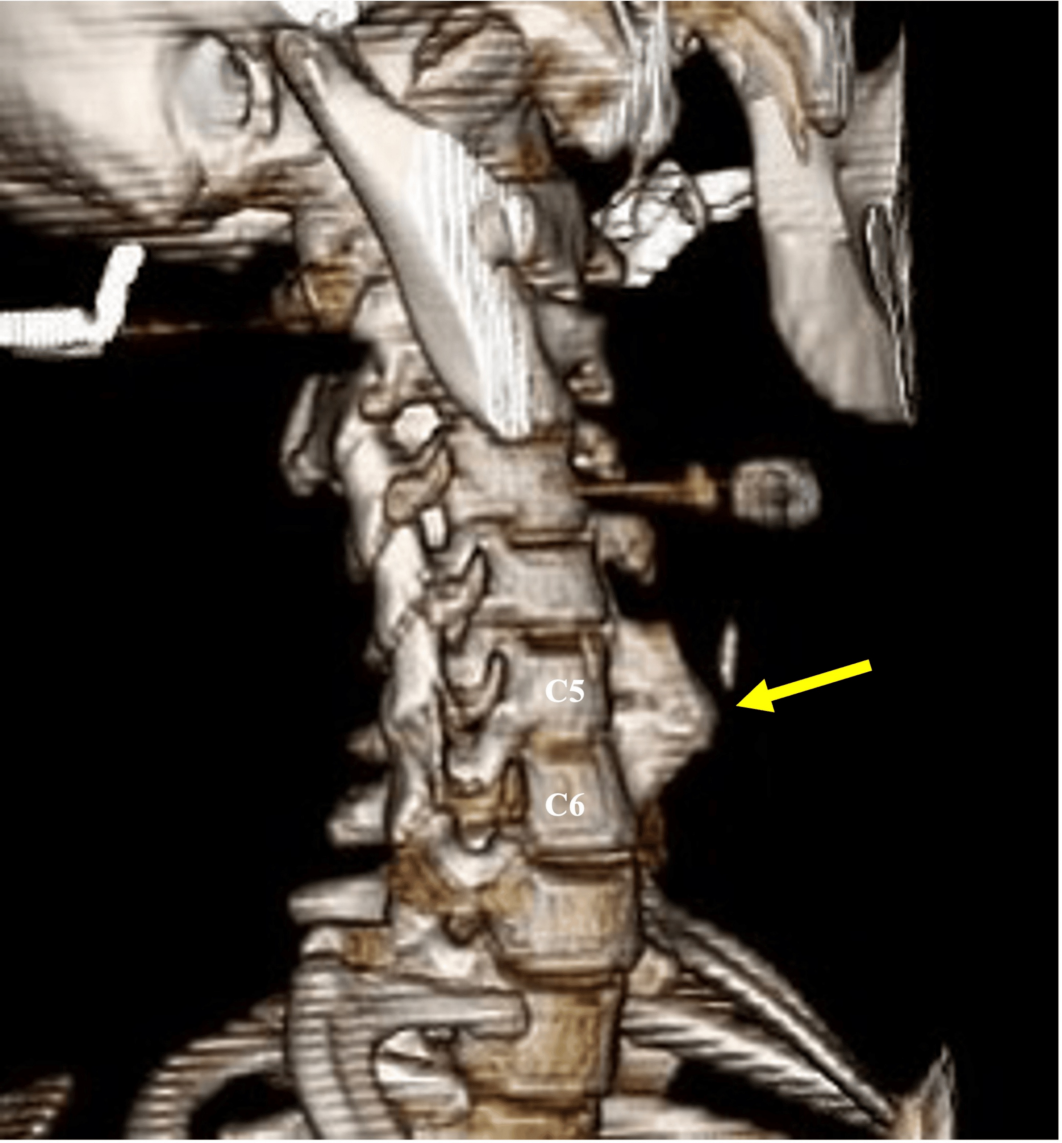
Three-dimensional surface-shaded display (SSD) reconstruction of the elongated left C5-C6 anterior tubercles of the transverse processes and accessory articulation. Anterior oblique view demonstrates the elongated left C5 and C6 anterior tubercles of the transverse processes and their accessory articulation (arrow). The normal morphology of the right-sided anterior tubercles of the transverse processes is visible for contralateral comparison. Note: SSD reconstruction is provided for supplementary three-dimensional spatial visualization only; all diagnostic interpretation, including assessment of cortical integrity, subchondral sclerosis, and transverse foraminal patency, was performed on the primary multiplanar reformatted images (Figure 1). A small metallic artifact is present on the right side of the image, which does not affect diagnostic interpretation. Volume rendering technique (VRT) was not available at the time of image acquisition through the teleradiology platform.

Based on the mechanism of injury, institution-specific protocols, and emergency physician clinical judgment, non-contrast CT of the cervical spine was performed (Canon Aquilion Prime SP, 160-slice; Canon Medical Systems, Otawara, Japan; 135 kVp with automatic dose modulation; 0.35-second rotation time; 2-mm contiguous axial images reconstructed in bone and soft tissue algorithms; 2-mm sagittal and coronal reformation in bone algorithm). Three-dimensional surface-shaded display (SSD) reconstructions were generated at the imaging site for illustrative purposes and were not employed in the primary diagnostic interpretation (Figures 1, 2). The specific clinical decision tool used to guide cervical spine imaging in this case was not available to the interpreting radiologist and is not documented in the imaging requisition.

The complete CT cervical spine interpretation systematically assessed all anatomical compartments evaluable on non-contrast CT, including osseous structures at all cervical levels from the craniocervical junction through the cervicothoracic junction, spinal alignment, intervertebral disc spaces, prevertebral soft tissues, the spinal canal, neural foramina, and transverse foramina. CT imaging demonstrated no acute fracture, subluxation, or listhesis. Prevertebral soft tissues were normal in thickness without evidence of hemorrhage or edema. The spinal canal was patent.

An incidental finding revealed unilateral elongated left C5-C6 anterior tubercles of the transverse processes with a well-corticated accessory articulation (Figures 1, 2). The elongated left anterior tubercles projected approximately 25 degrees anterolaterally from the sagittal vertebral body plane. Each elongated anterior tubercle measured approximately 10 mm in width and height and 10 mm in length as measured from the anterior cortex of the transverse foramen. The joint space width of the accessory articulation of the left C5-6 elongated anterior tubercles of the transverse processes measured 2 mm. The articulation exhibited smooth, sclerotic articular surfaces with a visible joint space, morphologically consistent with a synovial-type (diarthrodial) articulation, though definitive joint classification would require histological confirmation. No subchondral cysts, marginal osteophytes, or vacuum phenomena were identified. Transverse foramina were patent bilaterally at all cervical levels without osseous narrowing. Vertebral artery patency and caliber were not directly assessed on this non-contrast examination. The right-sided anterior tubercles at C5 and C6 demonstrated normal morphology. Costal elements were assessed bilaterally at all cervical levels (C3-C7), and no additional anterior tubercle elongation or cervical rib formation was identified, consistent with a congenital variant rather than an acute traumatic or degenerative process. No other congenital vertebral variants or anomalies were identified on this CT cervical spine.

No clinical or imaging follow-up was available, as this case was encountered in the acute trauma setting and interpreted via teleradiology without access to subsequent patient management or outcomes.

## Discussion

Elongation of the anterior tubercles of the cervical transverse process with accessory articulation formation is a rare anatomical variant first described in 1983 by Applbaum et al. [2]. The present case represents the sixth reported case at the C5-C6 level, the youngest patient documented with this variant, and expands the limited demographic spectrum of reported cases. A comparative summary of all reported cases with confirmed or probable accessory articulation formation is presented in Table 1.

**Table 1:** Reported cases of accessory articulation of elongated anterior tubercles of the transverse processes at the C5–C6 level Reported cases of accessory articulation of elongated anterior tubercles of the transverse processes at the C5-C6 level. All six cases demonstrated unilateral involvement and are listed chronologically by year of publication. Naidoo and Khan (2021) reported two cases in a single publication; only their C5-C6 case (Case 1, a 34-year-old male) is included [4]. Physical examination data and clinical follow-up for the present case were not available due to inherent limitations of the teleradiology practice setting. The present case represents the youngest reported patient with this variant. Abbreviations: CT, computed tomography; MDCT, multidetector computed tomography; MPR, multiplanar reformation; MVC, motor vehicle collision; SSD, surface-shaded display; VRT, volume rendering technique.

Parameter	Applbaum et al. (1983) [2]	Song et al. (2013) [6]	Unlu et al. (2015) [5]	Bilreiro et al. (2016) [7]	Naidoo & Khan (2021) [4]	Present Case
Journal	Skeletal Radiology	J Korean Soc Radiol	The Spine Journal	Surg Radiol Anat	BJR Case Reports	-
Age (Years)	32	36	56	40	34	22
Sex	Female	Male	Male	Female	Male	Female
Laterality	Right	Right	Right	Right	Left	Left
Presenting Symptoms	Numbness, tingling, weakness of the left upper extremity; hoarseness and globus sensation	Nuchal pain and left arm radiating pain (post MVC)	Chest pain, dyspnea, mild hoarseness (post MVC)	Frequent episodes of nonspecific neck pain	Asymptomatic (presented with facial fractures post-assault)	Asymptomatic; generalized pain (post MVC)
Clinical Context	Emergency department	Emergency department; car accident	Emergency department; traffic accident	Outpatient; symptomatic workup	Emergency department; mob assault	Emergency department; MVC; teleradiology
Variant Incidental	No	Yes	Yes	No	Yes	Yes
Physical &Neurological Exam	No neck masses; no muscular atrophy; normal radial pulse; no focal deficit	No neck masses or tender points; no muscular atrophy; normal radial pulse; no focal deficit	Not reported	Normal physical and neurological exam	Not reported	Not available (teleradiology)
Imaging Modality	Plain radiography, lateral tomography, and CT	Plain radiography, MDCT	Non-contrast CT	Plain radiography, non-contrast CT	CT (16-slice); MPR	Non-contrast CT (160-slice); MPR
3D Reconstruction	No	No	Yes (MPR and 3D)	Yes (VRT and MPR)	Yes (VRT)	Yes (SSD)
Associated Findings	Esophagram normal; no esophageal indentation	No fracture identified	Associated osteophytes; bilateral triticeous cartilages	No degenerative changes; no nerve compression signs	No associated cervical fractures	No degenerative changes; no cervical rib at C7
Treatment & Outcome	Conservative	Incidental; no treatment	Not reported	Symptomatic treatment	Not reported	No follow-up available
Key Differential Considered	Fracture, bone/cartilage tumor, osteophyte	Fracture, osteophyte	Osteochondroma, fracture	Fracture, osteophyte, bone tumor	Fracture	Fracture, osteophyte, bone tumor

The anterior tubercle of the cervical transverse process represents the embryological homologue of a rib at cervical levels, where Hox gene expression normally suppresses full rib development [2,14]. Elongation of this structure likely represents localized overdevelopment of the costal element at a level above C7, where it has developed more extensively than typical but has not formed a complete rib. This is analogous to the spectrum of transitional vertebral morphology observed at the lumbosacral junction, where costal elements may demonstrate varying degrees of development from rudimentary enlargement to complete rib formation. Ehara (1996) reported an association between elongated anterior tubercles and incomplete segmentation in the cervical spine, drawing a direct parallel to partial sacralization at the lumbosacral junction, where transitional morphology similarly reflects incomplete expression of a regional vertebral identity [10]. The formation of a discrete accessory articulation between adjacent elongated tubercles suggests that this developmental variant occurs sufficiently early in embryogenesis to permit joint formation, analogous to the costovertebral articulations formed by true ribs [15]. The unilateral presentation in the present case, as in the case reported by Bilreiro et al. (2016), remains embryologically unexplained and warrants further investigation [7]. The absence of a cervical rib at C7 indicates that the costal element overdevelopment was isolated to the C5-C6 level rather than representing a generalized shift in the axial formula. Costal elements were assessed bilaterally at all cervical levels (C3-C7) without additional abnormality.

The smooth, sclerotic articular surfaces with a visible joint space observed on CT are morphologically consistent with a synovial-type (diarthrodial) articulation, analogous to the costovertebral joints formed by true ribs. This classification remains a hypothesis based on imaging morphology. Definitive joint classification would require histological confirmation, which was not possible in this imaging-based case.

The differential diagnosis for a bony projection anterior to the cervical vertebral body on imaging includes several important considerations [2]. In the trauma setting, acute fracture fragments are the primary concern and can be distinguished by irregular, non-corticated margins and associated soft tissue injury [3]. Although misidentification of this variant as a fracture fragment is the most clinically consequential error in the trauma setting, this variant may also be misidentified as a degenerative osteophyte or neoplastic process, but less likely given the characteristic morphology. Osteophyte formation is common in older patients but typically occurs at vertebral body margins in association with degenerative disc disease. Bone tumors or exostoses would demonstrate different morphology and potentially aggressive features. Cervical ribs typically occur at C7 and articulate with the first thoracic rib rather than forming articulations between adjacent cervical vertebrae [13]. Key features distinguishing this congenital variant from acute pathology include well-corticated margins, smooth articular surfaces with sclerotic subchondral bone, and the presence of a defined joint space [2,3,6,7]. CT with multiplanar reformations provides detailed characterization [7].

The vertebral artery V2 segment typically enters the transverse foramen at C6 and ascends through C6 to C2, with the ventral rami of C5 and C6 coursing in the sulcus between the anterior and posterior tubercles of the transverse processes after exiting the intervertebral foramina [16-18]. In this case, the transverse foramina were patent bilaterally at all assessed cervical levels without osseous narrowing. On non-contrast CT, the bony transverse foramen can be assessed for osseous integrity, but the vertebral artery itself cannot be directly visualized or distinguished from the bony canal. The preliminary observation of no osseous foraminal compromise is therefore reassuring but does not constitute a definitive neurovascular assessment. CT angiography or magnetic resonance angiography would be required for direct evaluation of vertebral artery patency, caliber, and course. The elongated anterior tubercles project anterolaterally, approximately 25 degrees from the sagittal plane. Accessory muscles (e.g., scalenus minimus) and fibrous bands have been described in association with cervical costal element variants, but these soft tissue structures cannot be reliably assessed on non-contrast CT. MRI should be considered in future cases if symptoms potentially attributable to this variant develop, including radiculopathy, vascular insufficiency, or thoracic outlet-type symptoms, or if surgical intervention involving the anterior cervical spine is planned.

The clinical significance of this variant remains uncertain given its rarity. Prior reports have described both asymptomatic patients and those with neck pain or neurological symptoms, though establishing a causal relationship between the variant and symptoms is difficult given the confounding presence of trauma or other pathology in most reported cases [2,4-8]. The primary clinical concern is misidentification as an acute fracture fragment in the trauma setting, which could lead to an incorrect diagnosis of injury and inappropriate treatment [2,3,4-8]. Recognition of well-corticated margins and smooth articular surfaces is the key to correct identification [2,6,7]. Knowledge of variant anatomy is also relevant for cervical spine surgical planning, as transverse process variants may alter the expected relationship of the anterior tubercle to the vertebral artery and cervical nerve roots [18], although no surgical case involving this specific variant has been reported. 

## Conclusions

This case expands the limited literature on accessory articulation of elongated anterior tubercles of the cervical transverse processes and, to the best of the authors’ knowledge, represents the sixth reported case at the C5-C6 level and the youngest patient documented with this variant. Recognition of this rare anatomical variant is important for radiologists and emergency physicians to avoid misidentification as an acute fracture fragment in the trauma setting. Well-corticated margins, sclerotic articular surfaces, and a defined joint space help distinguish this congenital variant from acute pathology. CT with multiplanar reformations remains the primary modality for detailed characterization of this variant, including assessment of cortical integrity, joint morphology, and transverse foraminal patency. Future studies incorporating MRI correlation, quantitative morphometric analysis, and genetic evaluation may further clarify the embryological basis and clinical significance of this variant.
